# Unlocking potential of oxytocin: improving intracranial lymphatic drainage for Alzheimer's disease treatment

**DOI:** 10.7150/thno.98587

**Published:** 2024-07-15

**Authors:** Caihua Ye, Shengnan Wang, Lin Niu, Fan Yang, Guohe Wang, Siqi Wang, Jiamei Xie, Yihan Chen, Jinbo Qi, Hui Shen, Yan Dou, Junping Wang

**Affiliations:** 1 Department of Radiology, Tianjin Key Laboratory of Functional Imaging & Tianjin Institute of Radiology, Tianjin Medical University General Hospital, Tianjin 300052, P. R. China; 2 Department of Cellular Biology, School of Basic Science, Tianjin Medical University, Tianjin 300070, P. R. China.; 3 School of Life Sciences, Tianjin University, Tianjin300072, P. R. China.; 4 School of Medical Imaging, Tianjin Medical University, Tianjin 300203, China.

**Keywords:** Oxytocin, intracranial lymphatic system, Alzheimer's disease, cerebral hemodynamics, β-amyloid clearance, meningeal lymphangiogenesis

## Abstract

**Background:** The impediment to β-amyloid (Aβ) clearance caused by the invalid intracranial lymphatic drainage in Alzheimer's disease is pivotal to its pathogenesis, and finding reliable clinical available solutions to address this challenge remains elusive.

**Methods:** The potential role and underlying mechanisms of intranasal oxytocin administration, an approved clinical intervention, in improving intracranial lymphatic drainage in middle-old-aged APP/PS1 mice were investigated by live mouse imaging, ASL/CEST-MRI scanning, in vivo two-photon imaging, immunofluorescence staining, ELISA, RT-qPCR, Western blotting, RNA-seq analysis, and cognitive behavioral tests.

**Results:** Benefiting from multifaceted modulation of cerebral hemodynamics, aquaporin-4 polarization, meningeal lymphangiogenesis and transcriptional profiles, oxytocin administration normalized the structure and function of both the glymphatic and meningeal lymphatic systems severely impaired in middle-old-aged APP/PS1 mice. Consequently, this intervention facilitated the efficient drainage of Aβ from the brain parenchyma to the cerebrospinal fluid and then to the deep cervical lymph nodes for efficient clearance, as well as improvements in cognitive deficits.

**Conclusion:** This work broadens the underlying neuroprotective mechanisms and clinical applications of oxytocin medication, showcasing its promising therapeutic prospects in central nervous system diseases with intracranial lymphatic dysfunction.

## Introduction

Recently discovered intracranial lymphatic drainage system has been shed light on its crucial role in removing neurotoxic proteins such as β-amyloid (Aβ) from the brain, and its dysfunction is closely related to Alzheimer's disease (AD) 1, 2. This system is primarily composed of the classical glymphatic system and meningeal lymphatic system, which are responsible for the exchange of cerebrospinal fluid (CSF) with interstitial fluid (ISF) and meningeal lymphatic vessels (MLVs), respectively, and ultimately converge to cervical lymph nodes (CLNs) for clearance through the systemic circulation 3, 4. However, aging and degenerative diseases cause progressive structural and functional damage to the components of intracranial lymphatic system, resulting in obstructed or inefficient drainage, and eventually Aβ accumulation in the brain 5, 6. Therefore, considering its role as an intuitive mechanism for Aβ removal in the brain, targeting intracranial lymphatic system has emerged as a promising approach for treating AD based on central lymphatic clearance mechanism 7-9.

Indeed, the status of intracranial lymphatic drainage is highly susceptible to pathological disturbances during AD in various ways. Firstly, astrocyte dysfunction is recognized to be critical as it leads to the depolarization of aquaporin-4 (AQP4) expressed on their endfeet, creating severe obstacles to ISF-CSF exchange 10, 11. Secondly, cerebral hemodynamics can affect CSF activity, and its abnormalities in AD, such as reduced cerebral blood flow (CBF), can weaken the overall drainage and exchange efficiency 12, 13. Finally, chronic long-term damage to lymphatic endothelial cells would result in the blockage of the MLVs and the CLNs 7. Therefore, it is imperative to address perturbations in each of these pathways for efficient regulation of intracranial lymphatic drainage, posing an urgent need and great challenge for pharmacological intervention. Recently, certain biological factors and small molecule drugs have been revealed the potential to improve intracranial lymphatic drainage 14, 15. However, no clinically approved drugs have been proven so far.

Intranasal oxytocin (OT) administration is a clinically approved and effective means for assisting women with labor and lactation and has shown promising results in improving social disorders such as autism 16. As a neuroprotective peptide, OT has been reported to positively affect cognitive behaviors in many studies, including ours, with the most studied mechanism being its anti-inflammatory properties 17-19. Recent studies reveal that OT protects against neuronal damage by improving astroglial function in early stages of stroke 20. Interestingly, OT administration has been found to increase CBF both in normal rats and individuals at high risk for clinical psychosis 21, 22. Furthermore, many studies have confirmed that intranasal administration allows exogenous OT to reach the brain with high concentrations in the hippocampus and amygdala, providing the basis for its application in AD treatment 23-25. Despite these clues and hints, it remains unclear whether OT administration can regulate intracranial lymphatic drainage to facilitate Aβ clearance.

**Scheme 1:**
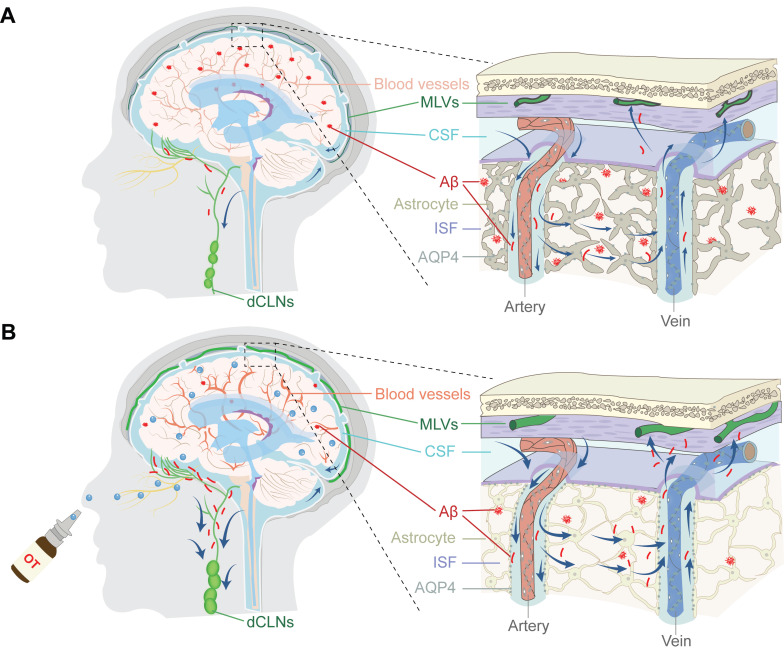
**Schematic representation of intracranial lymphatic drainage in AD and after OT administration**. (A) AD pathological status. In the glymphatic system, AQP4 depolarization results in reduced Aβ exchange between the ISF and CSF, while cerebral hemodynamic disorders exacerbate drainage inefficiency. In the meningeal lymphatic system, the damage to the MLVs causes occlusion of drainage to the dCLNs, leading to the inability to remove Aβ deposits from the brain. (B) Therapeutic effect of intranasal OT administration. Exogenous OT can enter the brain through the olfactory nerve bundle to simultaneously exert multiple regulatory functions, including enhancing cerebral hemodynamics, inhibiting AQP4 depolarization, and promoting lymphangiogenesis, ultimately restoring the structural integrity and Aβ clearance efficiency of intracranial lymphatic drainage system.

In this work, we unravel for the first time the potential role of exogenous OT administration in normalizing intracranial lymphatic drainage to ameliorate AD and elucidate the underlying mechanisms (**Scheme 1**). We find that intracranial lymphatic drainage is severely impaired in middle-old-aged but not young APP/PS1 mice, in which deep CLNs (dCLNs) play a crucial role. Here we provide evidence that intranasal OT administration is capable to significantly restore the structural integrity, the drainage efficiency, and Aβ clearance efficiency of intracranial lymphatic system, through simultaneous multiple regulatory mechanisms, including enhancing cerebral hemodynamics, inhibiting AQP4 depolarization, and promoting lymphangiogenesis. We also report the beneficial effect of OT on meningeal transcriptional profiles, associated with potential modulation of meningeal immunity-related pathways. This work expands the therapeutic scope of clinical OT medication, revealing for the first time its tantalizing promise for normalizing intracranial lymphatic drainage disorders in AD.

## Results

### Dysfunctional intracranial lymphatic drainage in middle-old-aged but not young APP/PS1 mice

**Figure 1:**
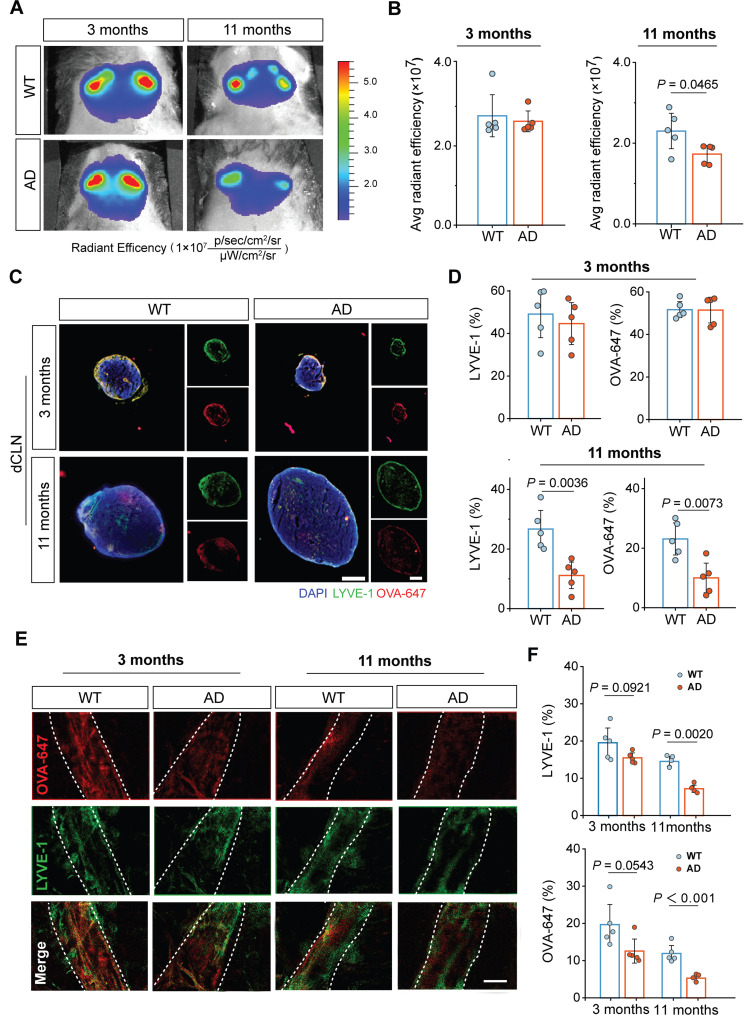
** Dysfunctional intracranial lymphatic drainage in middle-old-aged but not young APP/PS1 mice.** (**A**) *In vivo* brain ventral images of 3-month-old and 11-month-old APP/PS1 and WT mice at 2 h after i.c.m. injection of OVA-647. (**B**) Fluorescence quantification of OVA-647 in the cervical region of mice (*n* = 5). (**C**)**,** (**E**) Representative fluorescence images of LYVE-1 staining in (**C**) the dCLNs and (**E**) the meninges of mice at 2 h after i.c.m. injection of OVA-647. Scale bars, (**C**) 200 μm; (**E**) 100 μm. (**D**)**,** (**F**) Area fraction analysis of LYVE-1 and OVA-647 in (**D**) the dCLNs and (**F**) the meninges (*n* = 5). Data are presented as mean ± SD and analyzed by unpaired Student's t tests and one-way ANOVA followed by Bonferroni's post hoc test.

Currently, there is no evaluation study on the extent of intracranial lymphatic system impairments in APP/PS1 mice at different aging stages^7^. To investigate the difference in intracranial lymphatic drainage between middle-old-aged and young APP/PS1 mice, we first performed *in vivo* real-time fluorescence imaging to monitor the dynamic biodistribution of Alexa Fluor 647-conjugated ovalbumin (OVA-647) via the cisterna magna (i.c.m.) injection. Given that components in intracranial lymphatic system would eventually drain into the CLNs, fluorescence signals were detected in the cervical regions of mice at 2 h after injection (**Figure 1A**). No obvious changes in fluorescence intensity were observed between 3-month-old wildtype (WT) and APP/PS1 mice, but OVA-647 fluorescence intensities were significantly reduced in 11-month-old APP/PS1 mice compared with age-matched WT mice (**Figure 1B**).

To validate the structural impairments of the dCLNs and the MLVs in middle-old-aged but not young APP/PS1 mice, we distinguished and dissected the dCLNs and the dura mater for *ex vivo* immunofluorescence imaging by staining LYVE-1, a classic marker of lymphatic endothelial cells (**Figure S1 and Figure 1C**). No significant differences in LYVE-1 and OVA-647 coverage area ratios of the dCLNs were observed between 3-month-old APP/PS1 and WT mice, whereas both were significantly lower in 11-month-old APP/PS1 mice than in age-matched WT mice (**Figure 1C-D**). Similarly, there was only a decreasing trend of LYVE-1 and OVA-647 area coverage in the MLVs in 3-month-old APP/PS1 mice, while a marked reduction was found in 11-month-old APP/PS1 mice (**Figure 1E-F**). The common decrease in both LYVE-1 staining and injected OVA-647 retention suggested that impaired lymphatic structure leaded to weakened tracer influx into the MLVs and dCLNs, resulting in a significant reduction in drainage efficiency.

Collectively, these data suggested that intracranial lymphatic system and its drainage function were severely impaired in middle-old-aged but not young APP/PS1 mice.

### Improvement of cerebral hemodynamics and glymphatic drainage by OT administration in middle-old-aged APP/PS1 mice

**Figure 2:**
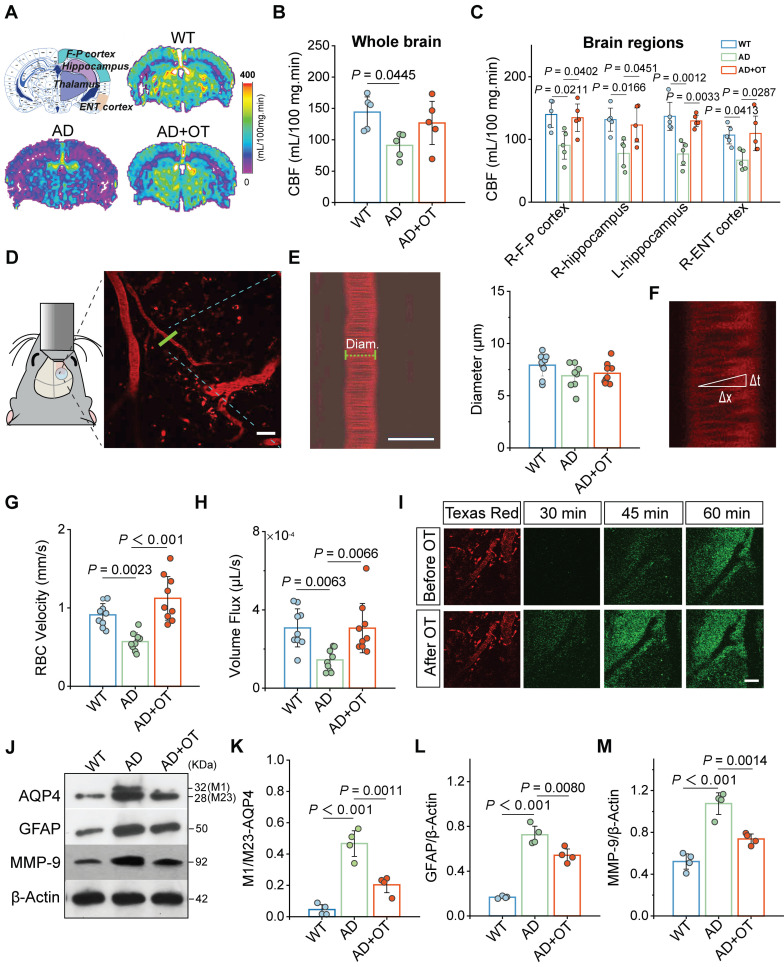
** Improvement of cerebral hemodynamics and glymphatic drainage by OT administration in middle-old-aged APP/PS1 mice.** (**A**) Coronal atlas of mouse brain anatomical template annotated with ROIs and representative ASL pseudo-color images of different groups. (**B-C**) CBF analysis in (**B**) whole brain and (**C**) specific brain regions (*n* = 5). (**D**) Visualization of penetrating arterioles 100 μm below the cortical surface by *in vivo* two-photon fluorescence angiography. Green lines, vertical line of the vessel axis drawn based on *X*-*T* line scanning. Scar bar, 20 μm. (**E**) Left, vessel diameter measurement. Scale bar, 10 μm. Right, vessel diameter quantification (*n* = 9). (**F**) Measurement of the distance (Δ*x*) and time (Δ*t*) of RBC movement along the intravascular scan line. (**G**) RBC velocity and (**H**) volume flux quantification (*n* = 9). (**I**) Time-lapsein *in vivo* two-photon imaging of CSF tracer influx into the cortex after i.c.m. injection in AD mice. Scale bar, 50 μm. (**J-M**) Representative immunoblots and quantitative analysis for (**K**) M1/M23-AQP4, (**L**) GFAP, and (**M**) MMP-9 (*n* = 4). Data are presented as mean ± SD and analyzed by one-way ANOVA followed by Bonferroni's post hoc test.

Previous studies reveal that adequate CBF provides critical support for rapid CSF transport throughout the brain, so insufficient blood supply to the AD brain could hinder the normal operation of intracranial lymphatic drainage 26. In view of this, arterial spin labeling (ASL) magnetic resonance imaging (MRI) was first performed to *in vivo* examine the effect of intranasal OT administration on CBF in 11-month-old APP/PS1 mice (hereafter referred to as AD mice). Representative ASL pseudo-color images presented marked CBF decrease in AD mouse brain compared with WT mice, which was surprisingly increased to near-normal levels after OT administration, both in the whole brain and in specific brain regions closely associated with memory and cognitive function, such as hippocampus, frontoparietal (F-P) and entorhinal (ENT) cortex (**Figure 2A-C**). In addition, there was also a certain upward trend of CBF in other brain regions, such as the thalamus, after OT administration in AD mice (**Figure S2**).

To further estimate the factors influencing CBF, fluorescence angiography was performed by intravenous injection of Texas Red-labeled Dextran-70 (TR-d70) using *in vivo* two-photon microscopy. According to branching direction, vessel diameter, and flow direction, penetrating arterioles were distinguished 100 μm below the cortical surface for vessel diameter measurement (**Figure 2D**). We found no significant changes in vessel diameter before and after OT administration in AD mice (**Figure 2E**), suggesting that the effect of OT on vessel constriction and dilation was almost negligible. Then, *X*-*T* line scans were acquired orthogonal to the vessel axis to record the distance and time of red blood cell (RBC) traversing along the vessel direction (**Figure 2F**). The calculated RBC velocity was significantly increased in AD mice after OT administration, accompanied by a marked improvement in vascular perfusion volume flux (**Figure 2G-H**). These results suggested that OT increased CBF by accelerating RBC velocity rather than dilating vessels.

To visualize the perivascular CSF drainage enhancement aided by CBF increase, fluorescent CSF tracer FITC-conjugated Dextran-70 (FITC-d70) was i.c.m. injected after vascular location through intravenous injection of TR-d70 in AD mice. As shown by *in vivo* two-photon fluorescence imaging, CSF tracer began to diffuse into the perivascular space along penetrating arterioles at 45 min after injection and entered the cortex at 60 min after injection (**Figure 2I**). After OT administration, CSF tracer spread as early as 30 min after injection and entered the cortex in large amounts at 45 min after injection. Moreover, green fluorescence intensity of CSF tracer diffused into perivascular space was also significantly enhanced after OT administration (**Figure 2I**). These results suggested that OT promoted CSF influx into the surrounding interstitium, which is highly favorable for CSF-ISF exchange.

Besides cerebral hemodynamics, astroglial water transport via AQP4 water channels also plays a vital role in the exchange between brain parenchyma and the CSF 27. It has been reported that AQP4 can be stably distributed as M23 subtype in astrocyte endfeet, which, once depolarization occurs, will be transformed into M1 subtype and transferred to the cell body, resulting in the restriction of CSF-ISF flow 28, 29. To investigate the impact of OT administration on AQP4 localization, Western blotting was conducted to reveal two distinct bands, the lower band (28 kDa) representing M23 isoform and the upper band (32 kDa) representing M1 isoform (**Figure 2J and Figure S3**). We observed that the increased M1:M23 ratio in AD mice was greatly decreased after OT administration (**Figure 2K**), indicating strong inhibition of AQP4 depolarization. Confocal immunofluorescence images of AQP4 staining in mouse brains clearly demonstrated a prevalence of tubular structures in WT mice (**Figure S4**), but instead became spheroid structures in AD mice, indicating a shift in AQP4 distribution from the endfeet to the cell body of astrocytes, suggesting depolarization. After OT administration, the number of tubular structures formed through AQP4 endfoot distribution was restored (**Figure S4**), further supporting the inhibition of AQP4 depolarization by OT. Meanwhile, OT administration also significantly inhibited the upregulation of total AQP4 expression in AD mice (**Figure S5**), indicating the attenuation of astrogliosis, further supported by similar level changes in glial fibrillary acidic protein (GFAP), a typical marker of astrocytes (**Figure 2L**). Since matrix metalloprotein 9 (MMP-9) mediated β-DG cleavage has been reported as a potential cause of AQP4 depolarization 30, we reasoned that improvement of aberrant AQP4 polarity by OT administration could be attributed to MMP-9 upregulation, validated by significantly declined levels after OT administration in AD mice (**Figure 2M**).

Taken together, these results uncovered that impairment of glymphatic drainage between brain parenchyma and the CSF in AD mice could be ameliorated by OT administration through the improvement of cerebral hemodynamics and AQP4 abnormal polarization.

### mprovement of meningeal lymphatic drainage by OT administration in middle-old-aged APP/PS1 mice

**Figure 3:**
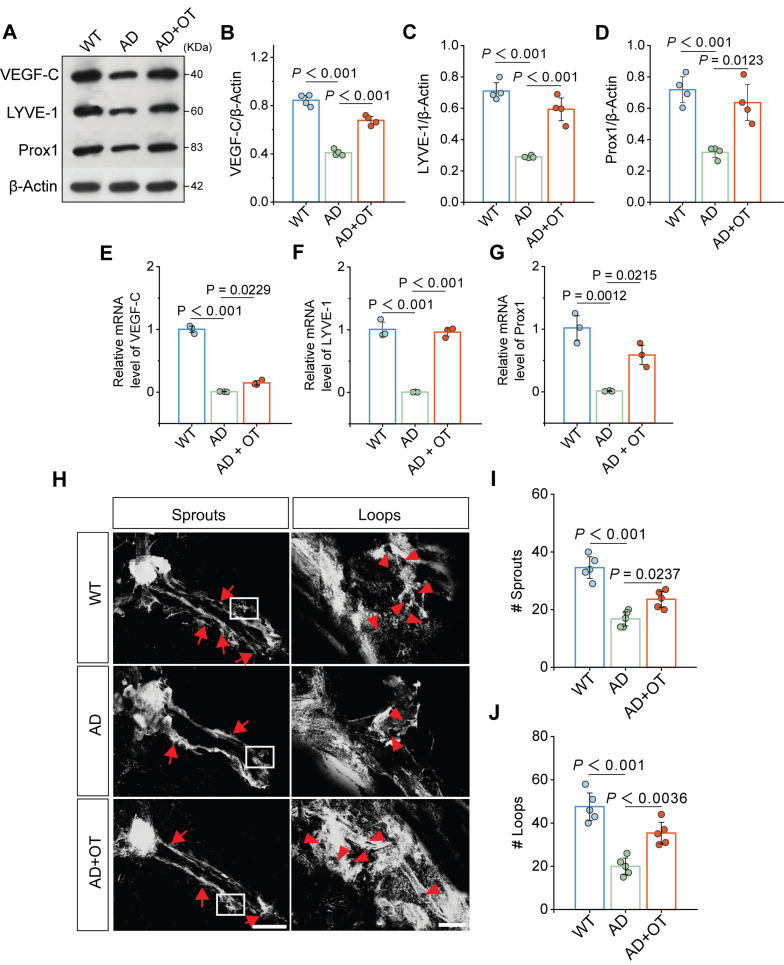
** Improvement of meningeal lymphatic structure by OT administration in middle-old-aged APP/PS1 mice.** (**A-D**) Representative immunoblots and quantitative analysis for (**B**) VEGF-C, (**C**) LYVE-1, and (**D**) Prox1 in the meninges (*n* = 4). (**E-G**) Quantitative analysis of the mRNA levels of (**E**) VEGF-C, (**F**) LYVE-1, and (**G**) Prox1 in the meninges by RT-qPCR (*n* = 3). (**H**) Representative confocal LYVE-1-stained images depicting MLV sprouts along the transverse sinuses (left) and MLV loops near lymphatic hotspots (right). Red arrow: the location of sprouts or loops. Scar bar, 1 mm (left) and 100 μm (right). (**I-J**) Quantification of the number of sprouts (**I**) and loops (**J**) in whole meningeal mounts (*n* = 5). Data are presented as mean ± SD and analyzed by one-way ANOVA followed by Bonferroni's post hoc test.

Since the MLVs directly constitute the meningeal lymphatic system, we verified the impact of OT administration on improving MLV structure. Western blotting showed that the expression of VEGF-C, an important regulator of MLV growth and maintenance, was greatly downregulated in AD mouse meninges and restored after OT administration (**Figure 3A-B and Figure S6**). As a result, the expression of LYVE-1 and Prox1, common markers of lymphatic endotheliocytes, was also significantly increased after OT administration (**Figure 3C-D**) 14, 31. RT-qPCR data also showed a significant increase in the mRNA levels of VEGF-C, LYVE-1 and Prox1 in AD mouse meninges after OT administration (**Figure 3E-G**), suggesting a beneficial effect of OT in inducing lymphangiogenesis. Furthermore, we examined the morphological changes of the MLVs by LYVE-1 immunofluorescence staining by a confocal microscopy. It was found that the complexity of the meningeal lymphatic vasculature was reduced in AD mice but increased after OT administration (**Figure 3H**). Quantitative analysis of the number of capillary sprouts and loops also showed a marked remodeling of MLV structure in AD mice after OT administration (**Figure 3I-J**), supporting the promotion of meningeal lymphangiogenesis by OT.

**Figure 4:**
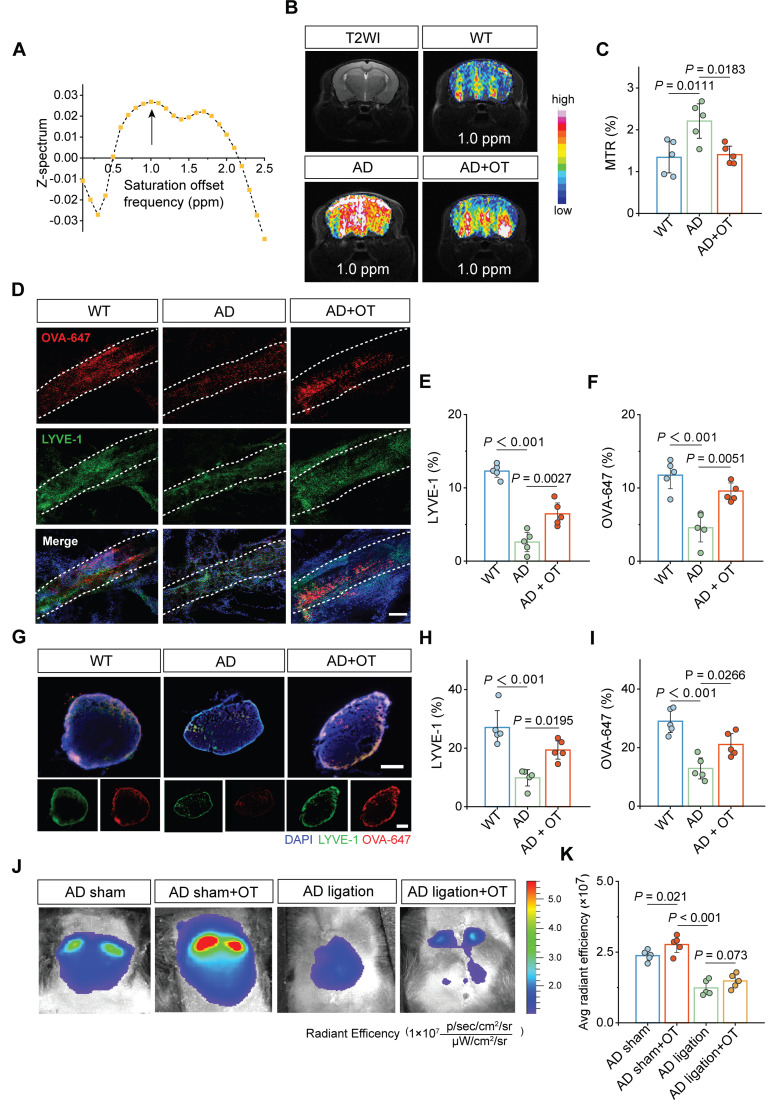
** Improvement of meningeal lymphatic drainage by OT administration in middle-old-aged APP/PS1 mice.** (**A**) Z-spectrum asymmetry curve of the whole mouse brain. Black arrows, 1 ppm location. (**B**) Representative T2-weighted (T2WI) and CEST images (MTR_asym_ maps at 1.0 ppm) of different groups. (**C**) MTR% analysis at 1.0 ppm in the whole brains (*n* = 5). (**D-G**) Representative fluorescence images of LYVE-1 staining in (**D**) the meninges and (**G**) the dCLNs of mice at 30 min after i.c.m. injection of OVA-647. Scale bars, (**D**) 100 μm; (**G**) 200 μm. (**E-F**)**,** (**H, I**) Area fraction analysis of LYVE-1 and OVA-647 in (**E, F**) the meninges and (**H, I**) the dCLNs (*n* = 5). (**J**) *In vivo* brain ventral images of AD mice with dCLN ligation at 30 min after i.c.m. injection of OVA-647. (**K**) Fluorescence quantification of OVA-647 in the cervical region of mice (*n* = 5). Data are presented as mean ± SD and analyzed by one-way ANOVA followed by Bonferroni's post hoc test.

To evaluate the role of MLV structural improvement on its drainage efficiency, chemical-exchange saturation-transfer magnetic resonance imaging (CEST-MRI) was used as an advanced tool to detect the level changes in trace lymphatic metabolites through saturation offset frequency relative to free water 32. Using 9.4T CEST-MRI, it was found that Z-spectrum asymmetry curve of mouse brain revealed a CEST peak centered at approximately 1.0 ppm (**Figure 4A**), based on which the representative images of the CEST effect was obtained (**Figure 4B**). Whole-brain CEST signal was significantly increased in AD mice compared to WT mice, indicating a marked accumulation of metabolites in AD brain that cannot be cleared. Encouragingly, OT administration substantially reduced the CEST signal, as shown in the magnetization transfer ratio (MTR) analysis (**Figure 4C**), implying the restoration of metabolite clearance and improvements in meningeal lymphatic drainage by OT administration.

Then, LYVE-1 staining of the dura mater was further performed after the i.c.m. injection of OVA-647. Confocal images showed significantly enhanced LYVE-1 area coverage in AD mice after OT administration, accompanied by a concomitant increase in OVA-647 area coverage (**Figure 4D-F**), suggesting promoted drainage function benefiting from improved lymphatic structure. Meanwhile, both LYVE-1 and OVA-647 area coverage in the dCLNs was also significantly increased after OT administration in AD mice (**Figure 4G-I**), illustrating the recovery of impaired structure and function of the dCLNs. To further confirm the critical role of the drain to the dCLNs, surgical ligation was conducted to physically block the afferent lymphatic vessels to the dCLNs (**Figure S7**), followed by *in vivo* fluorescence imaging of the cervical regions after the i.c.m. injection of OVA-647. We found that OT administration significantly enhanced fluorescence intensity in unligated AD mice (sham-operated group), while the fluorescence intensity of ligated AD mice was very low and could not be improved by OT administration (**Figure 4J-K**), indicating that the drain to the dCLNs was an indispensable link in the regulation of intracranial lymphatic drainage by OT.

Collectively, these data suggested that the promotion of lymphangiogenesis by OT administration improved meningeal lymphatic structure and drainage function in AD mice, in which the drain to the dCLNs played a central role.

### Enhancement of intracranial lymphatic clearance of Aβ by OT administration in middle-old-aged APP/PS1 mice

**Figure 5:**
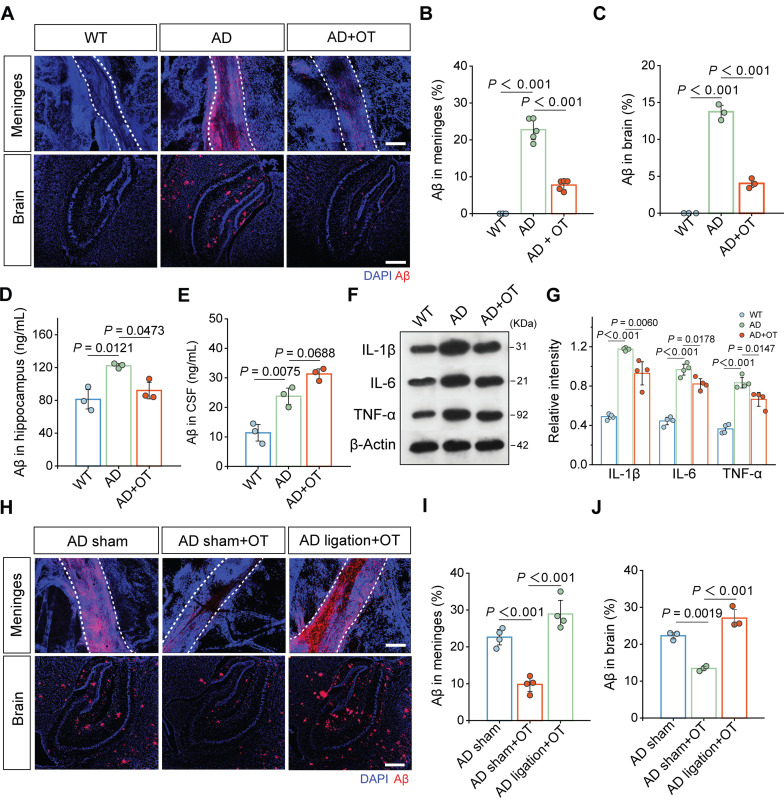
** Enhancement of intracranial lymphatic clearance of Aβ by OT administration in middle-old-aged APP/PS1 mice.** (**A**) Representative fluorescence images of Aβ staining in the meninges and brain sections. Scale bar, 100 μm. (**B**) Area fraction analysis of Aβ in the meninges (*n* = 5). (**C**) Area fraction analysis of Aβ in the cortex and hippocampus. (*n* = 3). (**D-E**) Levels of Aβ in the hippocampus and the CSF measured by ELISA (*n* = 3). (**F**) Representative immunoblots and quantitative analysis for (**G**) IL-1β, IL-6, and TNF-α (*n* = 4). (**H**) Representative fluorescence images of LYVE-1 and Aβ staining in the meninges and brain sections of mice with ligation. Scale bar, 100 μm. (**I**) Area fraction analysis of Aβ in the meninges (*n* = 4). (**J**) Area fraction analysis of Aβ in the cortex and hippocampus (*n* = 3). Data are presented as mean ± SD and analyzed by one-way ANOVA followed by Bonferroni's post hoc test.

Previous studies have shown that the accumulation of Aβ in the brain, the core pathological feature of AD, is considered the serious pathophysiological consequence of intracranial lymphatic dysfunction 33. To examine whether Aβ clearance could be enhanced by improvement of intracranial lymphatic drainage by OT administration, Aβ staining was performed on the dura mater and brain tissues. Substantial increase in Aβ distribution was found throughout the MLVs in AD mice, which was significantly reduced by OT administration (**Figure 5A-B**). Along with meningeal Aβ retention, we also observed the high level of Aβ burden in the brain parenchyma of AD mice, which was significantly reduced to the low level close to that of WT mice after OT administration (**Figure 5A and 5C**), further confirmed by quantitative measurement of hippocampal Aβ levels by ELISA analysis (**Figure 5D**).

Of note, Aβ levels in the CSF of AD mice was observed markedly increased (**Figure 5E**), which might be due to serious obstruction caused by impaired meningeal lymphatic drainage. Interestingly, OT administration further increased Aβ levels in the CSF (**Figure 5E**), which was attributed to improved efficiency of glymphatic drainage responsible for the brain parenchyma into the CSF. Combined with the reduction of Aβ levels in the brain parenchyma and the MLVs, we reasoned that OT promoted Aβ transfer from the brain parenchyma to the CSF to be cleared in time by the recovered MLVs. Due to the concern of the persistent pro-inflammatory activation of astrocytes by Aβ deposits, we also examined the effect of OT on alleviating neuroinflammation in the brain parenchyma. Elevated levels of typical inflammatory factors, such as IL-1β, IL-6, and TNF-α, were all significantly decreased in AD mouse brain after OT administration (**Figure 5F-G and Figure S8**), further supporting efficient clearance of Aβ through improved glymphatic drainage. To further corroborate the critical role of the drain to the dCLNs on Aβ clearance, we also observed changes in Aβ content and distribution after surgical ligation of the afferent lymphatic vessels to dCLNs. We found that the enhancement of Aβ clearance by OT administration was surprisingly counteracted after ligation, both in the MLVs (**Figure 5H-I**) and brain parenchyma (**Figure 5H and 5J**).

Collectively, these results demonstrated that the improvement of intracranial lymphatic drainage by OT administration facilitated efficient Aβ clearance from the brain in AD mice.

### Cognitive improvement in AD mice with intracranial lymphatic dysfunction by OT administration

Encouraged by the beneficial outcomes of OT administration on intracranial lymphatic drainage and Aβ clearance, we wondered whether OT treatment could ameliorate cognitive and memory deficits in AD mice. For treatment, 11-month-old AD mice with the aforementioned significant lymphatic drainage impairment received intranasal administration of OT every other day for 1 month, and a series of various behavioral paradigms were then employed to assess cognitive and memory abilities, including open field (OF), Y-maze, novel object cognization (NOR) and two-day water maze (WM) tests. To confirm that that OT could improve cognitive function by modulating intracranial lymphatic drainage, surgical ligation of the afferent lymphatic vessels of the dCLNs was performed in AD mice to assess behavioral performance after OT administration.

**Figure 6:**
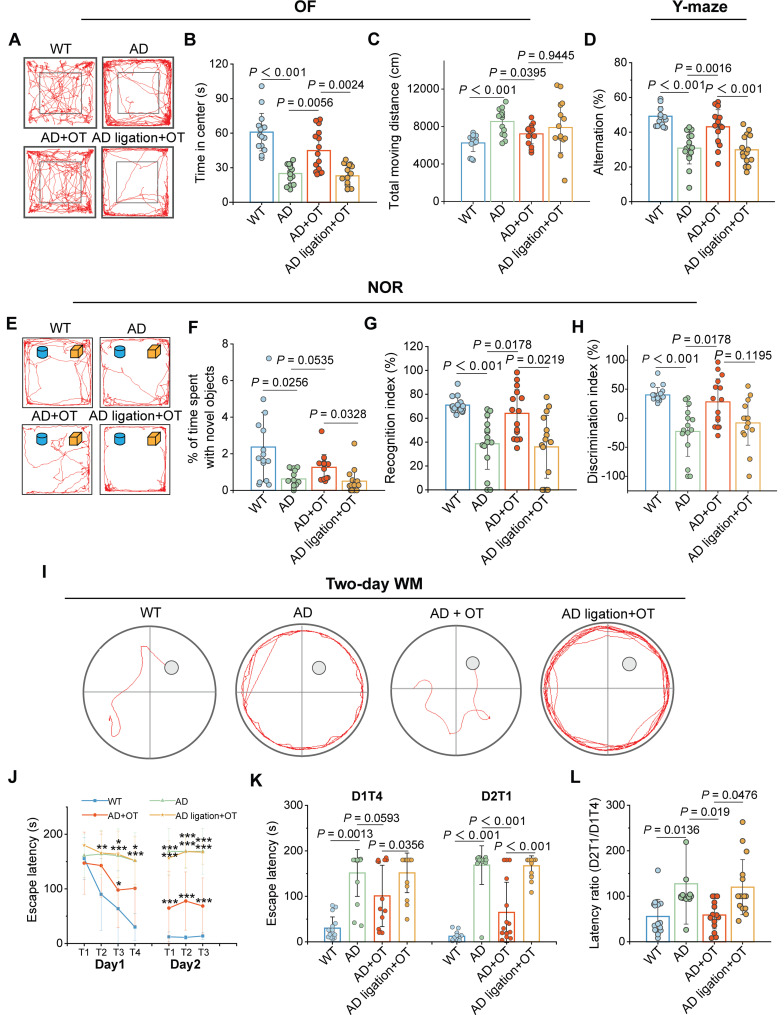
** Cognitive impairment in AD mice ameliorated by OT administration.** (**A**) Representative trajectories, (**B**) time spent in the center zone, and (**C**) total traveled distance in the OFT (*n* = 15). (**D**) Spontaneous alternation ratio in Y-maze test (*n* = 15). (**E**) Representative traces, (**F**) percentage of time spent with novel objects, (**G**) recognition index, and (**H**) discrimination index in the NOR test (*n* = 15). (**I**) Representative swimming paths in the hidden-platform probe phase of two-day WM test. (**J**) Escape latency for each trial in day 1 (visible trials) and day 2 (hidden trials), (**K**) escape latency analysis on day 1 trial 4 (D1T4) and day 2 trial 1 (D2T1), and (**L**) latency ratio by D2T1/D1T4 (*n* = 15). Data are presented as mean ± SD. *p < 0.05, **p < 0.01, ***p < 0.001, one-way ANOVA followed by Bonferroni's post hoc test.

Spontaneous locomotor function and anxiety-like behaviors of mice were examined in the OF test. Consistent with previous reports 34, the AD mice displayed a preference for the periphery or the corners of the arena rather than the center (**Figure 6A**), as indicated by a sharp decrease in time spent in the center zone (**Figure 6B**). Moreover, total moving distance of AD mice was much greater than that of WT mice (**Figure 6C**), suggesting the concomitant hyperkinesia during AD 35. In contrast, OT administration restored the motor performance of AD mice almost to normal levels observed in WT mice, but OT-administered ligated AD mice behaved similarly to AD mice (**Figure 6A-C**), suggesting the critical role of intracranial lymphatic drainage in the anxiolytic effect of OT.

In the Y-maze test for evaluating spatial working memory, AD mice exhibited greatly diminished spontaneous alternation ratio in three arms compared to WT mice, which was raised almost to normal levels after OT administration in AD mice rather than in ligated AD mice (**Figure 6D**). In the NOR test for assessing hippocampus-dependent object recognition memory, AD mice did not pay more attention to novel objects, whereas OT-treated mice obviously spent much more time on novel objects (**Figure 6E-F**). Both the recognition index and discrimination index for novel objects were significantly lower in AD mice compared to WT mice, but increased substantially after OT administration to almost the same levels as in WT mice (**Figure 6G-H**). However, above novelty memory improvement by OT administration was not observed in ligated AD mice (**Figure 6E-H**).

To evaluate hippocampus-dependent spatial learning and memory, we adopted a previously validated two-day WM protocol, including four visible-platform trials on day 1 and three hidden-platform trials on day 2 36. There was no significant difference in swimming speed among the groups **(Figure S9)**, so the interference with athletic ability of the mice could be excluded. During all trials on day 1, WT mice readily found the visible platform, but AD mice took significantly longer time to navigate to the platform location (**Figure 6J**). In contrast, escape latency was shortened after OT administration in AD mice, but this phenomenon was not observed in ligated AD mice (**Figure 6J**). On day 2, OT-treated AD mice found the platform as readily as WT mice, whereas OT-treated ligated AD mice spent a considerable time to reach the hidden platform, similar to AD mice (**Figure 6I and 6K**). Then, the ratio of the goal latency on the first trial of day 2 to that of the last trial of day 1 for each mouse (D2T1/D1T4) was calculated as an additional indicator of memory retention. The increased D2T1/D1T4 ratio in AD mice indicated poor cognitive performance compared to WT mice, but OT administration dramatically reduced this ratio, which was not observed under ligation conditions (**Figure 6L**).

Collectively, these finding strongly manifested that OT administration could ardently attenuate cognitive and memory deficits in AD mice with intracranial lymphatic dysfunction.

### Dysregulated meningeal transcriptional profiles restored by OT administration

**Figure 7:**
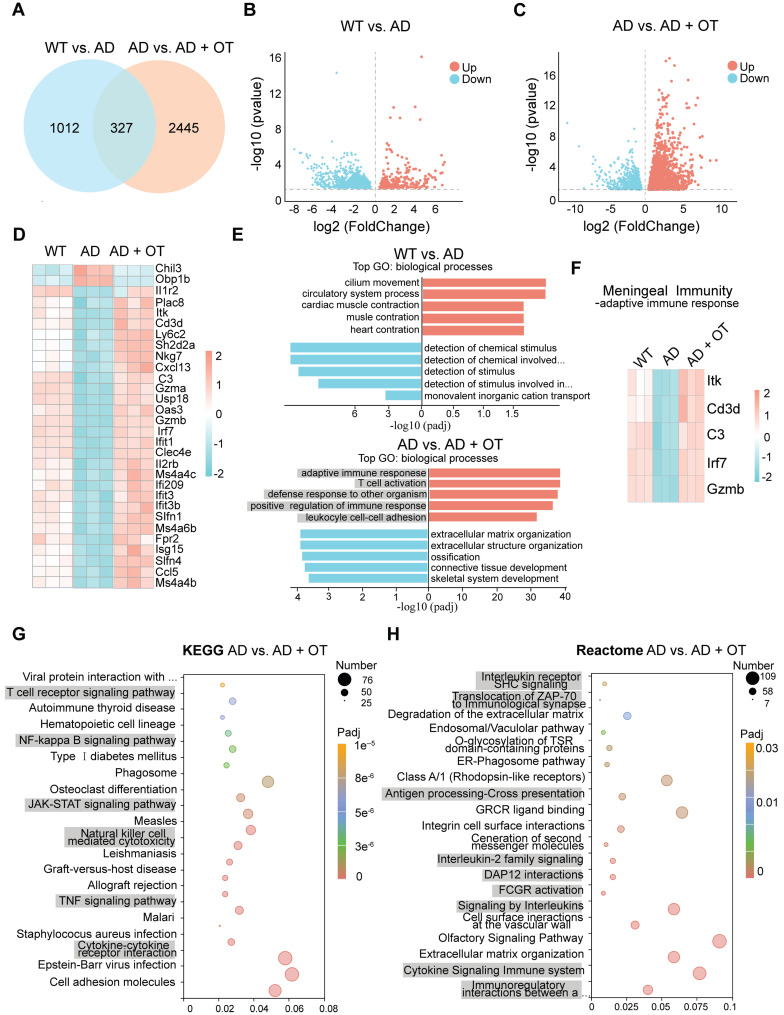
** Dysregulated meningeal transcriptional profiles restored by OT administration.** (**A**) Venn diagram showing intersection DEGs between the different comparison groups. (**B-C**) Volcano plots of significantly changed genes (two-fold change, *p <* 0.05). (**D**) Heatmap showing the top 30 DEGs in AD mice and altered after OT administration (*n* = 3). (**E**) The top five biological processes of significantly up- and down-regulated genes (*p <* 0.05). Red, GO terms for up-regulated genes; Blue, GO terms for down-regulated genes. (**F**) Expression of genes related to adaptive immune response among the top 30 DEGs (*n* = 3). (**G**) Scatter plots of the top 20 KEGG enrichment pathways in AD mice vs. OT-treated mice. (**H**) Scatter plots of the top 20 Reactome enrichment pathways in AD mice vs. OT-treated mice.

To gain insight into the beneficial effects of OT administration on overall meningeal function in AD mice, we conducted RNA sequencing (RNA-seq) based on the dura mater to analyze changes in the transcriptional landscape of the meninges. A total of 1339 differentially expressed genes (DEGs) were detected in AD mice compared with WT mice, with 297 genes being up-regulated and 1042 genes down-regulated (**Figure 7A-B**). After OT administration to AD mice, an even greater number of DEGs were identified, totaling 2772 genes with 1831 genes being up-regulated and 941 genes down-regulated (**Figure 7A-C**). These findings suggested that OT exerted a more pronounced impact on regulating gene expression in AD mice, particularly in terms of upregulation. And then, we identified the 30 most DEGs in AD mice compared with WT mice that were normalized after OT administration (**Figure 7D**). Of these DEGs, only the genes Chil3, Obp1b, and Il1r2 were downregulated after OT administration in AD mice, whereas the other DEGs were upregulated after OT administration.

It is noteworthy that among these upregulated genes, most were associated with biological processes involved in the meningeal immune responses. Gene Ontology (GO)-term enrichment analysis identified the top five upregulated and downregulated enriched biological processes in WT mice versus AD mice and AD mice versus OT-treated mice, respectively (**Figure 7E**). We found that the top 5 upregulated biological processes by OT administration included adaptive immune response, T cell activation, defense response to other organism, positive regulation of immune response, and leukocyle cell-cell adhesion, all of which were fundamental to meningeal immune homeostasis 8, 37. Among the top 30 DEGs, various genes associated with adaptive immune response, including lrf7, C3, Gzmb, ltk, and Cd3d, were down-regulated in AD mice but altered by OT administration (**Figure 7F**).

To uncover the underlying signaling pathways regulated by OT administration, we performed Kyoto Encyclopedia of Genes and Genomes (KEGG) pathway analysis and Reactome analysis in AD mice versus OT-treated mice. KEGG analysis revealed significant pathways related to meningeal immunity, mainly including T cell receptor signaling pathway, NF-κB signaling pathway, JAK-STAT signaling pathway, Natural killer cell mediated cytotoxicity, TNF signaling pathway, and Cytokine-cytokine receptor interaction (**Figure 7G**). Furthermore, Reactome pathway analysis showed enrichment in Interleukin receptor SHC signaling, Translocation of ZAP-70 to immunological synapse, Antigen processing-Cross presentation, Interleukin-2 family signaling, DAP12 interactions, FCGR activation, Signaling by interleukins, Cytokine signaling in immune system, and Immunoregulatory interactions between a lymphoid and a non-lymphoid cell (**Figure 7H**). According to previous reports 38, 39, all of these pathways mentioned above were closely associated with meningeal immunity.

Together, these findings preliminarily suggested that OT could exert a positive impact on overall meningeal transcriptional function through potential modulation of meningeal immunity-related pathways.

## Discussion

The discovery of intracranial lymphatic system has revolutionized traditional understanding of the brain's immune privilege, whose crucial role in eliminating pathological metabolic wastes holds promise for future advancements in neurological research and treatment options. This work uncovers for the first time the previously untapped potential of exogenous OT in normalizing intracranial lymphatic drainage to combat AD. We found that middle-old-aged but not young APP/PS1 mice exhibited markedly impaired intracranial lymphatic drainage. Encouragingly, our data showed that OT administration could enhance Aβ clearance from the brain through promoting glymphatic drainage from brain parenchyma to CSF and facilitating meningeal lymphatic drainage from CSF to the dCLNs, accompanied by improvements in cognitive and memory deficits in AD mice.

Here we investigated aging-related intracranial lymphatic drainage dysfunction in APP/PS1 mice, a readily available and well-established model for AD, but which has not been studied systematically before. We found prevalent impairments in both the glymphatic and meningeal lymphatic drainage in middle-old-aged (11-month-old) but not young (3-month-old) APP/PS1 mice, consistent with previous findings in 5XFAD mice 8. The CLNs have been identified as intermediary stations connecting intracranial and peripheral lymphatic systems, in which the dCLNs exhibit superior drainage capacity compared to the superficial CLNs (sCLNs) 41, 42. Our experimental results confirmed that the drain to the dCLNs was diminished in middle-old-aged APP/PS1 mice. In addition, dCLN volume was found to increase with age, regardless of AD occurrence, which might be due to multiple physiological changes associated with aging, including immune cell aggregation, increase or reorganization of stromal cells (such as fibroblasts) and supporting structures (such as lymphatic and vascular endothelial cells), and chronic low-grade inflammatory states 43-45.

Here we demonstrated for the first time that intranasal OT administration could improve intracranial lymphatic drainage in AD mice, in which the drain to the dCLNs played a vital role. Surgical ligation of the afferent lymphatic vessels to the dCLNs counteracted the enhancement of lymphatic drainage by OT administration, resulting in the failure to promote Aβ clearance and cognitive improvement. It should be noted that ligation significantly reduced but did not completely block drainage, suggesting the existence of alternative pathways for intracranial drainage out of the brain with weak effects. We further revealed the multifaceted underlying mechanisms by which OT exerts this effect, including cerebral hemodynamics, the structure and function of the glymphatic and meningeal lymphatic systems.

Our findings demonstrated that exogenous OT increases CBF in AD mice, which is consistent with previous discovery in normal rats and individuals at high risk for clinical psychosis 21, 46. The reason for this phenomenon has not been elucidated, so we further investigated changes in cerebral hemodynamic characteristics to reveal the potential causes. We found that OT has little impact on the vessel diameter but significantly increases RBC velocity. This suggests that the elevation in blood flow velocity may be a determining factor in the OT-induced CBF enhancement, rather than vasodilation. Our results were similar to previous studies of peripheral OT administration, which have showed that OT increased the heart rate and thus RBC velocity in pregnant women and rats 47, and long-term exposure to OT did not affect the diameter of cutaneous microvessels 48. Moreover, it has been suggested that OT can lead to dilation of small arterial vessels, while larger peripheral arteries may experience constriction 47, 49. This might be due to the fact that when OT acts on OT receptors in endothelial cells, it induces vasodilation by activating endothelial nitric oxide synthase 49; however, when OT acts on anti-diuretic hormone receptors on smooth muscle cells, it can promote vasoconstriction 50. Hence, the impact of OT on vascular diameter remains confused, which may be influenced by vessel size, receptor distribution, and tissue type, warranting further investigation in future studies.

The described effects on cerebral hemodynamics can be related to the promotion of intracranial lymphatic drainage by OT administration. Many studies indicate that CBF provides dynamic support for intracranial lymphatic drainage, and reduction in CBF could diminish the efficiency of intracranial lymphatic drainage 51, 52. For instance, stenosis of the cervical or intracranial arteries significantly reduces CBF, leading to impediments in intracranial lymphatic drainage 53. Furthermore, in the digoxin study, a transient deficiency in mice glymphatic function was found to parallel a decrease in CBF. Digoxin was shown to ameliorate glymphatic system damage by enhancing CBF, ultimately improving cognitive impairment in mice 54.

Aberrant AQP4 activation in astrocytes has been reported to have high relevance to the pathobiology of glymphatic drainage dysfunction. The ISF-CSF exchange has been identified to be mediated through the polarized AQP4 water channels located on the astroglial endfeet 55, 56. Once depolarization occurs, AQP4 channels migrate to the cell body and prevent effective exchange, which has been observed in various neurological disorders, including AD, Parkinson's disease, intracerebral hemorrhage, and transient focal ischemia 30, 57-59. Our data for the first time demonstrated a significant inhibition of AQP4 depolarization in AD mouse brains with intracranial drainage obstruction, following OT administration. To further reveal the regulatory mechanism of OT on AQP4 polarity, we evaluated the alteration of its upstream factor MMP-9, which can induce proteolytic cleavage of β-dystroglycan (β-DG), a key component controlling AQP4 anchoring to astroglial endfeet 60. As expected, the excessive MMP-9 levels in the brains of AD mice were significantly reduced by OT administration, suggesting that the inhibition of AQP4 depolarization by OT is likely to be achieved by blocking the MMP-9/β-DG/AQP4 pathways. A previous study confirmed that OT treatment suppressed brain MMP-9 expression in a stroke mouse model, which is somewhat consistent with our findings 54. Additionally, the alteration of AQP4 localization by OT might also be attributed to its direct inhibitory effect on astrogliosis, and which effect is dominant needs to be further studied.

Our discovery provided strong evidence that OT administration can promote lymphangiogenesis to improve the structural basis for meningeal lymphatic drainage in AD mice. Lymphatic endothelial cells are the core components of MLVs, and their functions can be regulated by a variety of signaling pathways, among which VEGF-C plays a central role 61-64. VEGF-C is reported to be essential for the development and maintenance of MLVs as a mitogen for vascular and lymphatic endothelial cells 61. Studies have shown that AAV-mediated VEGF-C expression enhances intracranial lymphatic drainage, thereby improving cerebral diseases such as stroke, traumatic brain injury and hepatic encephalopathy in mice 65-67. Despite the importance of VEGF-C, there is currently no direct evidence that OT can upregulate VEGF-C in MLVs. Our results showed for the first time that OT administration enhanced VEGF-C expression in AD mouse meninges, accompanied by upregulating the MLV markers LYVE-1 and Prox1. Quantitative analysis of capillary sprouts and loops also showed the increased complexity of meningeal lymphatic vasculature in AD mice after OT administration, further supporting the promotion of lymphangiogenesis by OT.

Besides structural effects, improvements in overall meningeal function by OT may also have beneficial implications on drainage efficiency. Recent reports have shown that the meninges possess a diverse population of immune cells whose functional status profoundly affects central nervous system homeostasis, thus drawing increasing attention to meningeal immunity 68. Manipulating meningeal immunity has been proven to mitigate the damage to meningeal lymphatic function, thereby alleviating neurological deficits such as multiple sclerosis, traumatic brain injury, and AD 69. Our RNA-seq data based on the dura mater revealed most of the DEGs in AD mouse meninges were enriched in dysregulated pathways such as adaptive immune response, T cell activation and other defense responses, which were observed to be restored after OT administration. Although macroscopic analysis at the tissue level failed to reflect lymphatic molecular features, these pathways have been reported inseparable from the maintenance of meningeal immune homeostasis 37, 70. Furthermore, RT-qPCR data based on the dura mater revealed the similar changes in mRNA levels of VEGF-C, LYVE-1, and Prox1, suggesting the regulatory effect of OT on the transcriptional profiles of meningeal lymphatic system.

Although our results suggested that OT administration is effective in improving intracranial lymphatic drainage, we cannot completely rule out the involvement of other therapeutic mechanisms in AD treatment due to the wide range of biological effects of OT, such as direct regulation of neuroinflammation 17, 18. Moreover, the positive effects of OT on blood uptake or microglial phagocytosis may also be responsible for Aβ clearance, and further clarification of CSF drainage throughout the brain and Aβ content in the dCLNs would be helpful. Another limitation of this study is that molecular lymphatic features were suppressed by tissue-level RNA-seq analysis covering multiple biological processes. It would be useful to further isolate meningeal lymphatic endothelial cells and focus on changes in their transcriptional profiles, numbers, and functional markers. In future studies, attention should be paid to optimizing anesthetic methods to minimize influence on hemodynamics and CSF flow, and larger sample sizes are expected to validate these findings. Furthermore, it is worth considering whether the effects of OT on CSF efflux to the nasal-associated lymphatic system have an impact on nasal delivery of OT into the brain. In addition, it will be of particular interest to explore whether OT also regulates the intracranial lymphatic system under normal physiological conditions.

## Conclusion

In summary, our study sheds light on the feasibility and multifaceted underlying mechanisms of OT administration in normalizing intracranial lymphatic drainage in middle-old-aged APP/PS1 mice. We found that exogenous OT can realize efficient Aβ clearance from the brain, which might be due to its positive effects on the structure and function of both glymphatic and meningeal lymphatic systems, including the improvements in cerebral hemodynamics, AQP4 polarization, meningeal lymphangiogenesis and transcriptional profiles, thus greatly restoring cognitive function in AD mice. If our preclinical results are confirmed in further clinical trials, OT administration-mediated intracranial lymphatic recovery can be used as a clinically applicable therapeutic approach for AD and other diseases with intracranial lymphatic disorders.

## Methods

**Mice and administration.** 3-month-old C57BL/6J mice and APP/PS1 mice were purchased from Beijing HFK Bioscience Co., Ltd., and 11-month-old C57BL/6J mice and APP/PS1 mice were purchased from Shanghai Model Organisms Center, Inc. Mice were maintained and bred in-house under standard housing conditions (23 ± 2°C, 12 h/12 h light/dark cycles and fed ad libitum). All animal experiments were performed in accordance with the guidelines approved by the Animal Care and Use Committee of the Institute of Radiation Medicine, Chinese Academy of Medical Sciences (IRB2023-DWFL-166). For administration, APP/PS1 mice were randomly selected and intranasally administered with 20 μL OT (4.2 μg/μL) every other day for a total of 15 times. Meanwhile, APP/PS1 mice and age-matched C57BL/6J mice were administered saline, serving as the group of AD mice and WT mice, respectively.

**i.c.m. injections.** Mice were anesthetized with isoflurane, and their heads were secured in a stereotaxic instrument. The dorsal neck skin was shaved and cleaned with iodine and 70% ethanol. Ophthalmic solution was applied to the eyes to prevent drying. The skin and neck muscles of mice were dissected to expose the cisterna magna. Subsequently, OVA-647 (Invitrogen; O34784; 5 μL; 1 mg/mL in artificial CSF) and FITC-70kDa (Shanghai Maokang Biochnology Co., Ltd.; MS0905; 5 μL; 2 mg/mL in artificial CSF) were respectively injected into the CSF-filled cisterna magna compartment using a Hamilton syringe coupled to a 33-gauge needle. After injection, the needle was kept in place for an additional 5 min to avoid backflow. The mice were then sutured and allowed to recover on a heating pad before regaining consciousness.

**Lymphatic vessel ligation.** In brief, mice were anesthetized with isoflurane, and the skin of the neck was shaved and cleaned with iodine and 70% ethanol. A midline incision was made 4 mm superior to manubrium of the sternum. The trachea and sternocleidomastoid muscles were retracted to expose and distinguish the dCLNs on each side. The afferent lymphatic vessels on each side were ligated with 10-0 synthetic, non-absorbable sutures. Sham-operated mice underwent a sham surgery, which included the skin incision and retraction of the sternocleidomastoid muscle only. The skin was then sutured, and the mice were allowed to recover on a heatpad until fully awake. Subsequently, EB dye (Sigma; E2129; 5μL; 10%) was injected into the CSF-filled cisterna magna compartment using a Hamilton syringe coupled to a 33-gauge needle. After 30 minutes, EB staining was observed to confirm successful ligation.

**Live mouse imaging.** The cervical skin of mice was shaved to minimum the imaging interference from auto-fluorescence of the animals. OVA-647 (5 μL; 1 mg/mL in artificial CSF) was i.c.m. injected into mice at a rate of 1 μL/min. Fluorescence intensity was measured using an *in vivo* fluorescence imaging system (IVIS Spectrum, PerkinElmer) at 2 h after injection. Briefly, the ROI containing the anatomical range of the CLNs was drawn manually at least three times in duplicate, and the average fluorescence intensity within the ROI was automatically measured by the instrument's built-in software. For ligation of apparent lymphatic vessels to the dCLNs, imaging was conducted 30 min post-injection. The dCLNs were harvested at the indicated timepoints and then analyzed for immunofluorescence quantification.

**Immunofluorescence imaging.** The dCLNs, brain, and skull were fixed with 4% PFA overnight and dehydrated using a sucrose gradient. Then, the complete skull was transferred into sterilized water for additional 2 days. These fresh-frozen dCLNs sections (10 μm thick), and brain sections (20 μm thick) were sliced using a cryostat (Leica). Under a stereomicroscope, the dura mater was carefully dissected from the skull using a pair of ophthalmic forceps and then unfolded on glass slide. The prepared fresh-frozen dCLNs sections, brain sections and meningeal whole-mounts were washed with PBS and incubated with 0.5% Triton X-100 for 5 min, followed by incubation in 0.5% bovine serum albumin (BSA) for 1 h at room temperature. This blocking step was followed by incubation with appropriate dilutions of primary antibodies overnight at 4°C: rat anti-LYVE-1 (Santa Cruz, sc-65647, 1:300), rabbit anti-β-amyloid (Cell signaling, clone D54D2, 1:500) and rabbit anti-AQP4 (abclone, A11210, 1:200). Goat anti-rat Alexa Fluor 488 IgG antibodies (Bioss, bs-0293G-AF488, 1:600), goat anti-rabbit Alexa Fluor 594 IgG antibodies (ZSGB-BIO, ZF-0513, 1:200) and goat anti-rabbit Alexa Fluor 488 IgG antibodies (ZSGB-BIO, ZF-0511, 1:200) were applied for 1 h in the dark at room temperature. After washing three times for 5 min with PBS, the tissue was incubated for 5 min with DAPI. Preparations were stored at 4°C for no more than one week until images were acquired using a confocal microscopy (Leica Stellaris 5, Germany) and an inverted fluorescence microscope (Olympus, Japan). Quantitative analysis of the acquired images was conducted using ImageJ software. The area fraction was assessed by dividing the area coverage by LYVE-1, Aβ and OVA-647 fluorescence over the DAPI-stained area of the dCLNs, brain, and meningeal whole-mounts.

**ASL-MRI scanning.** MRI scans were performed using a 9.4T MRI scanner (Bruker BioSpec 94/30 USR, Germany). During the scanning process, the mice were anesthetized with inhaled isoflurane, and the heart rate and breathing were monitored using an animal physiological monitoring system. T2-weighted images were acquired using a fast spin echo rapid acquisition relaxation enhanced (RARE) (TurboRARE) sequence with the following parameters: repetition time (TR) = 2500 ms, echo time (TE) = 33 ms, field of view (FOV) = 20 mm × 20 mm, slice thickness = 0.5 mm, and number of slices = 19. These T2-weighted images were used to align the subsequent arterial spin labeling magnetic resonance imaging (ASL) location. ASL images were obtained from echo-planar imaging-fluid-attenuated inversion recovery (EPI-FLAIR) sequences with acquisition parameters of TR = 10000 ms, TE = 20.11 ms, FOV= 20 mm × 20 mm, slice thickness = 1 mm.

The workstation software ParaVision 6.0 (Bruker Corporation) was employed to reconstruct CBF images for ASL analysis. For quantitative CBF analysis, ROIs were manually outlined on the CBF map, following the mouse brain atlas by Paxinos and Watson. Concurrently, T2‑weighted anatomical images with the same slice location were displayed for reference. The ROIs were carefully drawn to include the bilateral F-P cortex, hippocampus, thalamus, ENT cortex and the entire brain. Based on the CBF map, the average blood flow within the ROIs of each brain region was calculated.

**CEST-MRI scanning.** Magnetic field homogenization (shimming) was performed on the main magnetic field (B0). For the CEST imaging experiments, the scan parameters for the animals were as follows: TR = 6000 ms, TE = 5.93 ms, FOV = 20 × 20 mm^2^, slice thickness = 1 mm, matrix = 96 × 96. The Z-spectrum extended from -5 to 5 ppm with an interval of 0.25 ppm.

The raw CEST scan results were exported from the workstation to MATLAB (Matlab R2016a, Mathworks, Natick, MA, USA) running under Windows 10 for post-processing. The saturation signal (S_sat_) was normalized by the unsaturated signal (S_0_) to generate the Z-spectrum. The Z-spectrum was then corrected for B_0_ nonuniformity. The MTR signal was obtained by subtracting the z-spectrum signal at the corresponding negative offset (-Δω) from that at the positive offset (Δω). We calculated the CEST effect using the following equation:



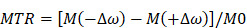



The hippocampus, responsible for cognition and learning functions, is the primary region affected in the early stages of AD. Therefore, we selected the most extensive section of the hippocampus, as shown in echo-planar images featured in a high-resolution MRI atlas, as our designated CEST slice.

***In vivo* two-photon imaging.** For pretreatment, mice were secured on the stage of a stereotaxic device for cranial window surgery after anesthesia. A 5-mm diameter craniotomy was drilled in the skull, 1.5 mm lateral and 1.5 mm posterior to bregma. The dura mater was covered with a glass coverslip, and a custom perforated metal plate was attached to the perimeter of the cranial window using adapted acrylic adhesive. Mice were intraperitoneally injected daily with antibiotics (ceftriaxone sodium: dexamethasone = 8: 1) after surgery, with the dosage gradually reduced until imaging was performed seven days later.

To visualize the blood vessels, 100 μL of TR-d70 (molecular weight, 70 kDa; 1% in saline; Invitrogen) were intravenously injected, which is impermeable to the blood-brain barrier and requires immediate observation after injection. First, the cerebral vascular network was imaged using 512 × 512 pixels, spanning from the brain surface to a depth of 200 μm using Nikon software. To evaluate vascular diameter (D, mm), 2-3 blood vessels were selected for each subject. Using ImageJ software, line-scan images were employed to draw perpendicular lines spanning from one side of the blood vessel wall to the other, facilitating the measurement of length.

To calculate RBC velocity, the dark bands corresponded to the movement of RBC along the vessel's length, and the steepness of the lines indicated the speed at which hemoglobin was traversing. The line-scan images were constructed with the distance (x, mm) of the measured blood vessel on the x-axis and the scan time (t, s) on the y-axis. Blood flow velocity (v, mm/s) was determined using the formula v = Δx/Δt. Ten lines were measured to calculate the average blood flow velocity (mm/s). Vascular perfusion volume, which represents the volume of blood passing through the cross-section of the blood vessel per unit time, was calculated by the following formula: 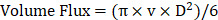.

To visualize the CSF-ISF influx, FITC-70kDa (5 μL; 2 mg/mL in artificial CSF) as CSF tracer was immediately i.c.m. injected at a rate of 1 μL/min following vascular imaging. Tracer movement within the perivascular space was recorded using dual-channel (FITC and Texas Red) 512 × 512 pixels image acquisition at 30 min, 45 min and 60 min. Subsequently, the mice were given intranasal administration of OT. Based on the location of the superficial artery, the same cortical layer was identified, and imaging was conducted at 30 min, 45 min and 60 min. Image analysis was performed using ImageJ software.

**OF test.** Mice were acclimated to the behaviour room for at least 30 min before commencing the test. The mice were placed in the center of opaque white plastic box (40 cm × 40 cm) and allowed 5 min in the arena, during which their exploration was recorded and analyzed using Smart v3.0 video tracking software (RWD Life Science Co., Ltd, China). The center zone was defined as a region in the center measuring 12.5 × 12.5 cm^2^. The arena was cleaned with 70% ethanol after each run to remove odor interference.

**NOR test.** Mice were introduced into the same apparatus in OF test containing two identical objects A and B (with the same color and shapes), where they could explore freely for 5 min. After an interval of 1 h, one of the old object B was substituted with a new object C (with different color and shapes) and mice were again placed in the arena for 5 min. Exploration of an object was recorded when a mouse approached and touched the object with its nose or forepaws. The recognition index was defined as [TC / (TA + TC) ×100%] and the discrimination index was defined as [(TC - TA)/ (TA + TC) ×100%], where TA and TC indicated time spent exploring familiar object A and novel object C, respectively. After each run, the arena was sanitized with 70% ethanol to eliminate any interference from odors.

**Y-maze test.** The Y-maze, made of dark polyvinyl plastic, consists of three arms with equal angles of 120°. Each arm is 35 cm long, 5 cm wide and 10 cm high. The mice were placed in the center of the arena and allowed to explore for 5 min. The alternation score for each mouse was calculated according to the following equation: spontaneous alternation (%) = [(number of alternations) / (total arm entries - 2)] × 100. Before each test, the box was cleaned with 70% alcohol to eliminate the odour and waste of mice.

**Two-day WM test.** The two-day WM test protocol consisted of a training phase (Day1) and test phase (Day2) as previously reported 36. The test was performed using a circular tank (diameter, 120 cm; height, 50 cm) containing water at a temperature of 21 ± 1°C, which was divided into four equal quadrants and featured a platform (8 cm in diameter) located in the center of the northwest quadrant. On the first day, the platform was elevated 1 cm from the water level, and each mouse was introduced to the pool on four occasions (4 trials) with a one-hour gap between each trial. On the second day, the platform was submerged 1 cm beneath the water's surface, and each mouse was subjected to the pool three times (3 trials) with a one-hour interval between each trial. The mice were released with their orientation towards the wall, and each attempt lasted a maximum of 180 s. Escape latency was recorded and analyzed with a computerized video tracking system (SMARTSUPER, Panlab, Spain).

**CSF collection.** According to previously published studies, the mice were fully anesthetized and tightly secured in a stereotaxic frame to ensure that the head was at a 120° angle to the neck 40. The skin of the dorsal neck was shaved and cleaned with iodine and 70% ethanol. Using scissors and curved forceps, the muscle over the base of the skull was carefully dissected away, exposing the dura mater over the cisterna magna (which is triangular in shape with 1-2 large blood vessels visible in this area). Using the tip of a glass capillary, the membrane was punctured until resistance was encountered, allowing CSF to be automatically drawn into the capillary tube. The CSF samples were then centrifuged at 2000g for 10 minutes to eliminate any blood materials. After centrifugation, the CSF was promptly stored at -80°C for further analysis.

**ELISA.** The concentration of Aβ peptides in the hippocampus and CSF of different treatment groups were determined using the Mouse Aβ ELISA Kit (ml037203, Shanghai Mlbio Biotechnology Co., Ltd.). Hippocampus tissues were weighed and then ground in PBS (tissue weight (g): PBS volume (mL) = 1:9) with a protease inhibitor by Precellys Evolution Super Homogenizer (Bertin, France). For the CSF, 5 μL CSF was diluted in 45 μL of sample diluent. Then, according to the manufacturer's instructions, the absorbance at 450 nm was measured using a Bio-Rad 680 microplate reader (Biotek, USA).

**RT-qPCR.** The fresh mouse dura mater was flash-frozen on dry ice and then stored in a -80°C freezer. Total RNA was extracted using an RNA Isolation kit (AG21023, Accurate Biology Co., Ltd.) according to the manufacturer's instructions, and used for cDNA synthesis using designed primers and reverse transcriptase. The following primers were used: LYVE-1 forward: GCCAACGAGGCCTGTAAGAT; LYVE-1 reverse: TCCAACCCATCCATAGCTGC; VEGF-C forward: 5'-TGTGCTTCTTGTCTCTGGCG-3'; VEGF-C reverse: 5'-CCTTCAAAAGCCTTGACCTCG-3'; Prox1 forward: 5'-CGTGAAGTTCAACAGATGCATTA-3'; Prox1 reverse: 5'-CAAAGTCATTTGCTTTGTTGTAGTG-3'. RT-qPCR was performed using GADPH as an internal reference. Relative mRNA expression levels were analyzed through the 2^ΔΔCt^ method.

**Western blot.** Brain tissues and the dura mater were respectively collected from mice after various treatments. Total protein was extracted, and the protein concentration was determined using a BCA protein assay kit. Then, equal volumes of protein were loaded onto 10-12% SDS-polyacrylamide gel electrophoresis gels and transferred to polyvinylidene difluoride membranes. After blocking, the membranes were incubated with the following primary antibodies at 4°C overnight: anti-AQP4, anti-GFAP, anti-MMP-9, anti-IL-1β, IL-6 and anti-TNF-α used in brain tissue studies and anti-VEGF-C, anti-LYVE-1 and anti-Prox1 used in meningeal studies. The primary antibody dilutions and sources were shown in **Table S1**. β-Actin were used as an internal control. Then, the membranes were incubated with secondary antibody, immersed in chemiluminescence detection solution, and exposed to radiographic film. Images were obtained using Image Lab software (Bio-Rad, UAS), and the gray values of the protein bands were analyzed using ImageJ software.

**RNA-seq analysis.** The mouse dura mater was immediately stripped from the skulls of euthanized mice, quickly frozen in dry ice, and stored at -80°C until further use. The RNA extraction, library preparation, and RNA sequencing were commissioned by Novogene (Beijing, China). Briefly, total RNA samples were assessed for the purity, concentration, and integrity prior to further analysis according to Agilent 2100 bioanalyzer. Purified mRNA was fragmented and reverse transcribed to create the final cDNA library in accordance with the protocol for the mRNA-Seq sample preparation kit (Illumina, San Diego, USA). The cDNA libraries were then sequenced based on effective concentration and target data volume. Finally, statistical methods were used to compare the differences in gene expression between two or more conditions, identify specific genes related to the conditions, and further analyze the biological significance of these specific genes. The analysis process included data quality control, mapping to the reference genome, gene expression quantification, difference expression analysis, and functional analysis.

**Statistical analysis and reproducibility.** Statistical analyses were conducted using SPSS25 software. All data exhibited a normal distribution, as determined by the Shapiro-Wilk normality test, and were presented as mean ± SD. Two-tailed unpaired Student's t-test was used for two-group comparisons. One-way analysis of variance (ANOVA) followed by Bonferroni's post hoc test was used for multiple-group comparisons. Values of *p* < 0.05 were considered as statistically significant (*p < 0.05, **p < 0.01, ***p < 0.001).

## Supplementary Material

Supplementary figures and tables.

## Abbreviations

qRT-PCR: quantitative real-time PCR
VEGF-C: vascular endothelial growth factor-C
LYVE-1: lymphatic vessel endothelial receptor-1
Prox1: prospero homeobox protein 1
IL-1β: interleukin-1β
IL-6: interleukin-6
TNF-α: tumor necrosis factor-α
ELISA: enzyme-linked immunosorbent assay
cDNA: complementary DNA
