# Supplementary material for: Unlocking potential of oxytocin: improving intracranial lymphatic drainage for Alzheimer's disease treatment
Source: Theranostics. 2024 Jul 15;14(11):4331–51. doi: 10.7150/thno.98587 (PMC11303076; doi:10.7150/thno.98587)
Supplement: Supplementary file 1 — Supplementary figures and tables. [file thnov14p4331s1.pdf]

Supplementary Material

For

Unlocking Potential of Oxytocin: Improving Intracranial Lymphatic Drainage for Alzheimer's Disease Treatment

Caihua Ye,[1,#] Shengnan Wang,[1,#] Lin Niu,[2,#] Fan Yang,[3] Guohe Wang,[4] Siqi Wang[1], Jiamei Xie,[1] Yihan Chen,[1] Jinbo Qi,[1] Hui Shen,[2,] Yan Dou,[1,]* and Junping Wang[1,]**

[1]Department of Radiology, Tianjin Key Laboratory of Functional Imaging & Tianjin Institute of Radiology, Tianjin Medical University General Hospital, Tianjin 300052, P. R. China

[2]Department of Cellular Biology, School of Basic Science, Tianjin Medical University, Tianjin 300070, P. R. China.

[3]School of Life Sciences, Tianjin University, Tianjin300072, P. R. China.

[4]School of Medical Imaging, Tianjin Medical University, Tianjin 300203, China.

[#] These authors contributed equally to this work.

*E-mail: douyan@tmu.edu.cn; wangjunping_tj@163.com; shenhui@tmu.edu.cn

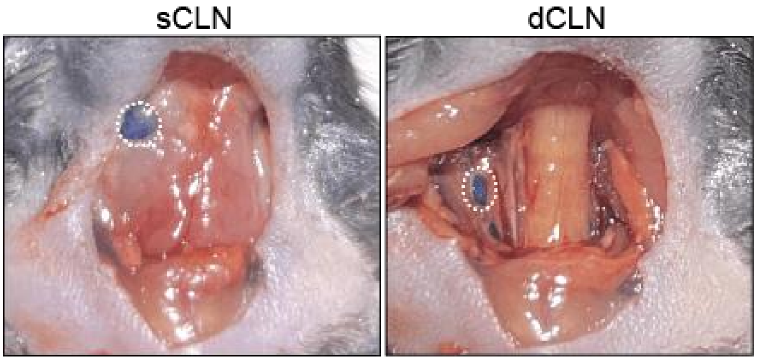

Figure S1. Representative images of the anatomical locations of superficial and deep CLNs.

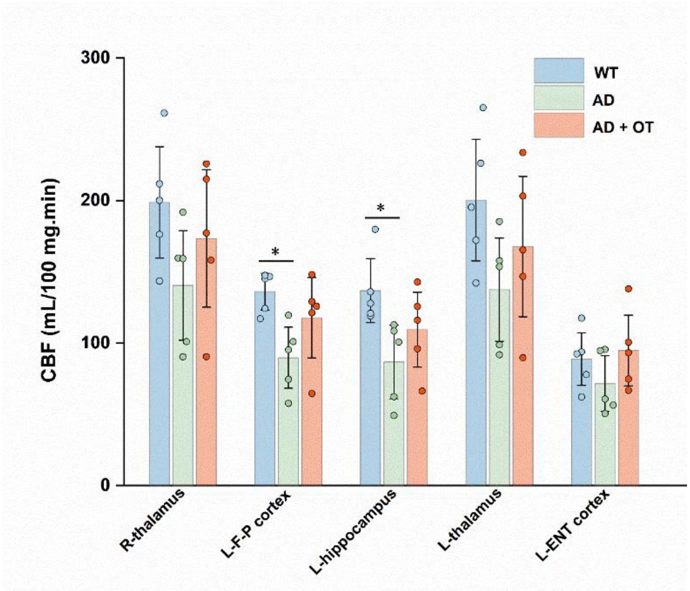

Figure S2. Quantitative analysis of CBF in specific brain regions (n = 5). Data are presented as mean ± SD.

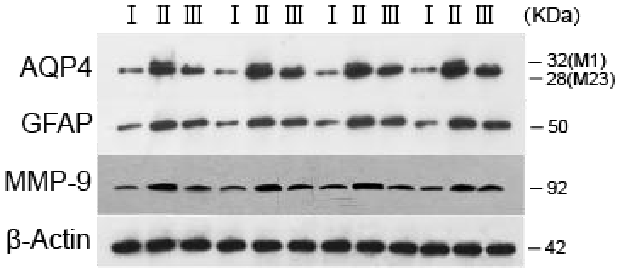

Figure S3. Representative immunoblots for AQP4, GFAP, and MMP-9 (n = 4). Group number: I: WT; II: AD; III: AD + OT.

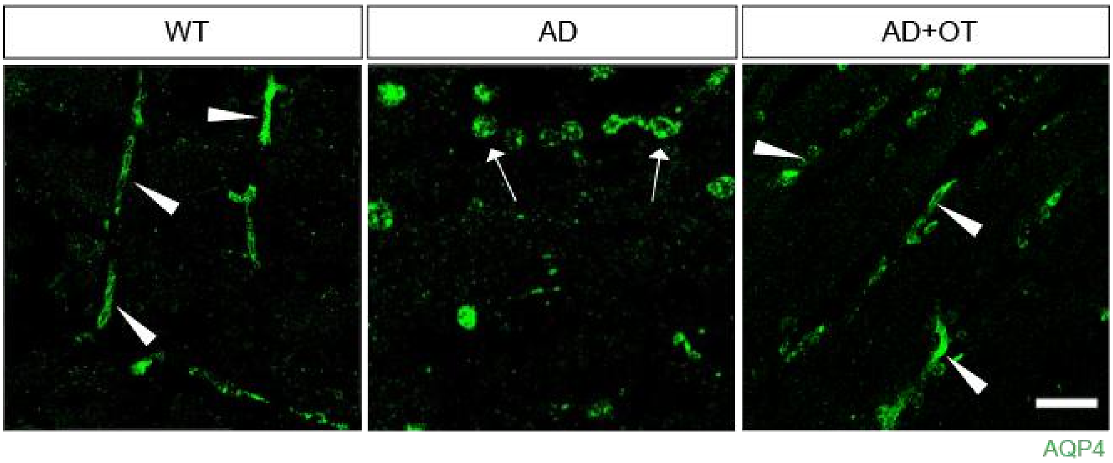

Figure S4. Immunofluorescence staining of AQP4 in mouse brain. Scale bars, 20 μm.

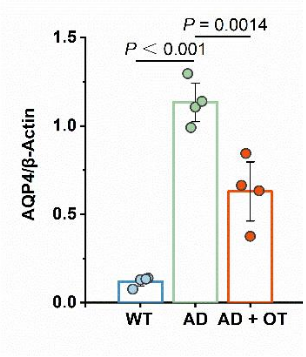

Figure S5. Quantitative analysis of the expression levels of AQP4 (n = 4) by Western blot in Figure 2j. Data are presented as mean ± SD.

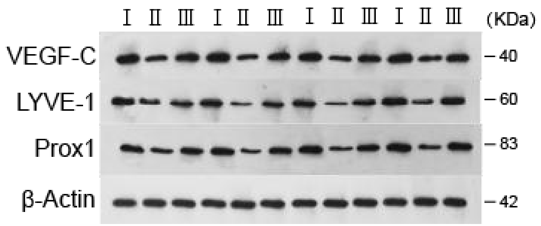

Figure S6. Representative immunoblots for VEGF-C, LYVE-1, and Prox1 (n = 4). Group number: I: WT; II: AD; III: AD + OT.

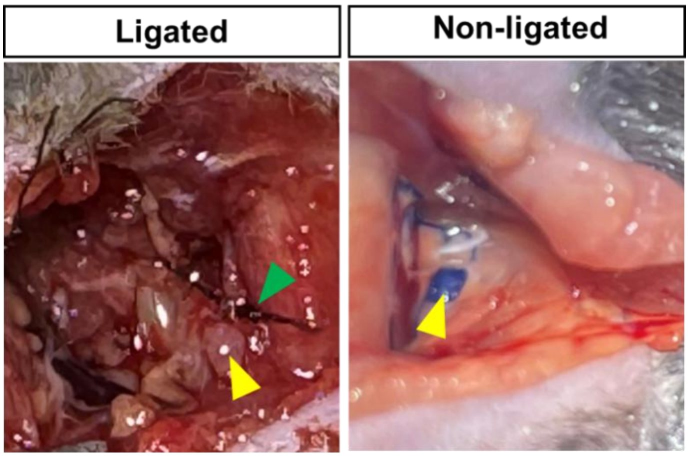

Figure S7. Representative images of the ligation and non-ligation surgery. Yellow: the node location; Green: the ligation site.

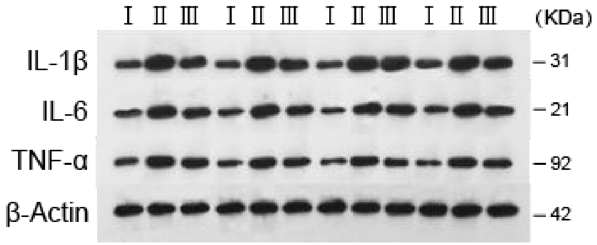

Figure S8. Representative immunoblots for IL-1β, IL-6, and TNF-α (n = 4). Group number: I: WT; II: AD; III: AD + OT.

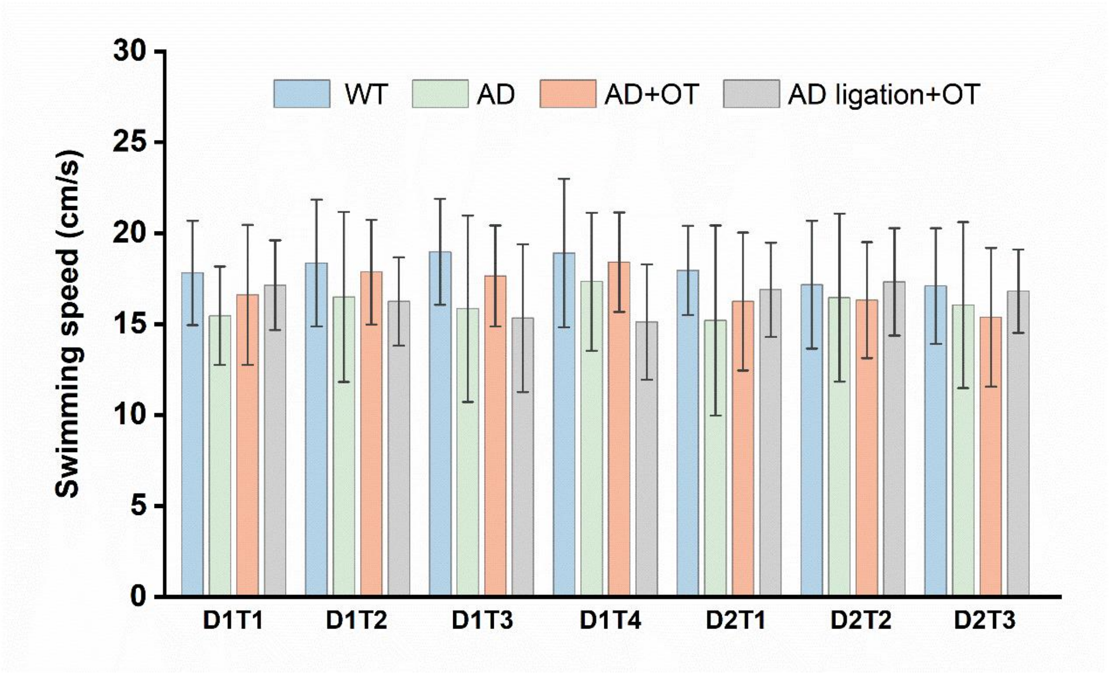

Figure S9. Swimming speed of mice in two-day water maze test (n = 15). Data are presented as mean ± SD.

Table S1. The dilution ratio and source of primary antibodies used in this study.

Antibodies Type	Dilution	Source	Catalog
AQP4	1:150	Santa	sc-32739
GFAP	1:200	Santa	sc-33673
MMP-9	1:300	Santa	sc-393859
IL-1β	1:800	Abcam	ab9722
IL-6	1:800	Abcam	ab6672
TNF-α	1:800	Abcam	ab9739
VEGF-C	1:200	Santa	sc-374628
LYVE-1	1:200	Santa	sc-65647
Prox1	1:200	Santa	sc-81983
β-Actin	1:800	ZSGB-BIO	TA-09
HRP-conjugated goat anti-rabbit IgG	1:3000	ZSGB-BIO	ZB-2305
